# Imaging characteristics of osteitis condensans ilii. A follow-up study

**DOI:** 10.1007/s00256-026-05360-7

**Published:** 2026-09-07

**Authors:** Anne Grethe Jurik, Alexander Andrew Matthew Mills, Amalie Birk Niesen, Emma Marie Mills, Rosa Marie Kiil, Anna Zejden, Asta Linauskas

**Affiliations:** 1 Department of Rheumatology, North Denmark Region Hospital, Hjoerring, Denmark; 2 Department of Radiology, Aarhus University Hospital, Palle Juul-Jensens, Boulevard 99, 8200 Aarhus N, Denmark; 3 Department of Clinical Medicine, Aarhus University, Aarhus, Denmark; 4 Department of Clinical Medicine, Aalborg University, Aalborg, Denmark

**Keywords:** Osteitis condensans ilii, Magnetic resonance imaging, Radiography, Axial spondyloarthritis, Diagnosis, Differential

## Abstract

**Objective:**

To evaluate the diagnostic ability of combined clinical and MRI findings in a cohort of patients initially diagnosed with axial spondyloarthritis (axSpA) and to detect potential patients with osteitis condensans ilii (OCI) for further analyses.

**Materials and methods:**

We conducted a retrospective follow-up study of 129 consecutive patients aged ≥ 18 years (49 women/80 men) diagnosed with axSpA during the period 2012–2023. Uniform baseline MRI of the sacroiliac joints (SIJ) and spine with concomitant radiography and/or CT were reassessed by an experienced radiologist using fulfillment of thresholds for SIJ MRI changes consistent with axSpA and a global assessment of all imaging material*.* Clinical and biochemical findings at baseline and during follow-up were reevaluated and in combination with imaging findings used as a reference standard for axSpA and OCI changes, respectively. Imaging findings consistent with OCI were confirmed by a second experienced radiologist and OCI imaging characteristics were systematically assessed.

**Results:**

axSpA was confirmed in 95 patients. Seven women had OCI and 27 (15 women/12 men) had non-specific SIJ bone marrow edema (BME). None of the OCI women fulfilled radiographic criteria for axSpA, but all fulfilled fat deposition thresholds on MRI consistent with axSpA and five also fulfilled thresholds for BME. The detected OCI MRI characteristics were as follows: sclerosis predominating at the anterior-middle iliac joint areas with BME and fat deposition at the periphery, and subchondral BME and fat deposition predominantly in the sacrum.

**Conclusion:**

The location of sclerosis, BME, and fat deposition can be used to diagnose OCI changes on MRI.

## Introduction

Axial spondyloarthritis (axSpA) is a chronic inflammatory disease affecting the sacroiliac joints (SIJ) and/or the spine. The diagnosis of axSpA can be challenging both clinically and by imaging. Other conditions may simulate sacroiliitis by imaging and can be accompanied by low back and buttock pain [1–3]. Biochemical tests may have limited value; although human leukocyte antigen subtype B27 (HLA-B27) is frequent in axSpA, the prevalence of axSpA in HLA-B27 positive individuals is only approx. 5% and C-reactive protein (CRP) is inadequate to distinguish active axSpA from other conditions [4, 5].

MRI plays a pivotal role in the diagnosis of non-radiographic axSpA and increases the diagnostic findings in radiographic axSpA (i.e. ankylosing spondylitis), being able to detect signs of active inflammation presenting as subchondral bone marrow edema (BME) as well as structural changes in the form of erosions, subchondral sclerosis, fat deposition and joint space alteration. However, similar changes, especially BME, are also seen in other conditions, e.g., due to pregnancy, osteitis condensans ilii (OCI) and load-related and/or degenerative changes [1]. Especially OCI can present MRI changes simulating axSpA sacroiliitis and is an important axSpA differential diagnosis if accompanied by pain [2, 3]. There is no universally accepted definition of OCI changes by MRI although a longitudinal study of pregnancy-related sclerosis supported a sclerosis depth ≥ 8 mm to be indicative of OCI [6]. The current knowledge of manifest OCI MRI features is based on three retrospective studies, all describing subchondral iliac sclerosis to be frequently accompanied by BME, whereas erosions were rare [7–10].

Data comparing OCI MRI changes with radiographic changes are limited, although OCI originally was described and defined based on radiographic appearance encompassing unilateral or bilateral triangular-shaped iliac sclerosis at the lower portion of the SIJ, spared SI joint space and absence of erosions [11–14]. Further definitions of radiographic characteristics are lacking. The estimated prevalence of radiographic OCI in general populations is between 0.9% and 2.5% with a female predominance [13, 14]. The changes can be asymptomatic incidental findings, but may be accompanied by pain, observed in 2.5–8.9% of patients presenting with low back or buttock pain [2, 3, 15].

As OCI can both clinically and on MRI simulate sacroiliitis, the objectives of this study were to evaluate the diagnostic ability of combined clinical and MRI findings in a cohort of patients initially diagnosed with axSpA, and to detect possible OCI patients and assess their clinical and imaging features.

## Materials and methods

### Participants

**Fig. 1:**
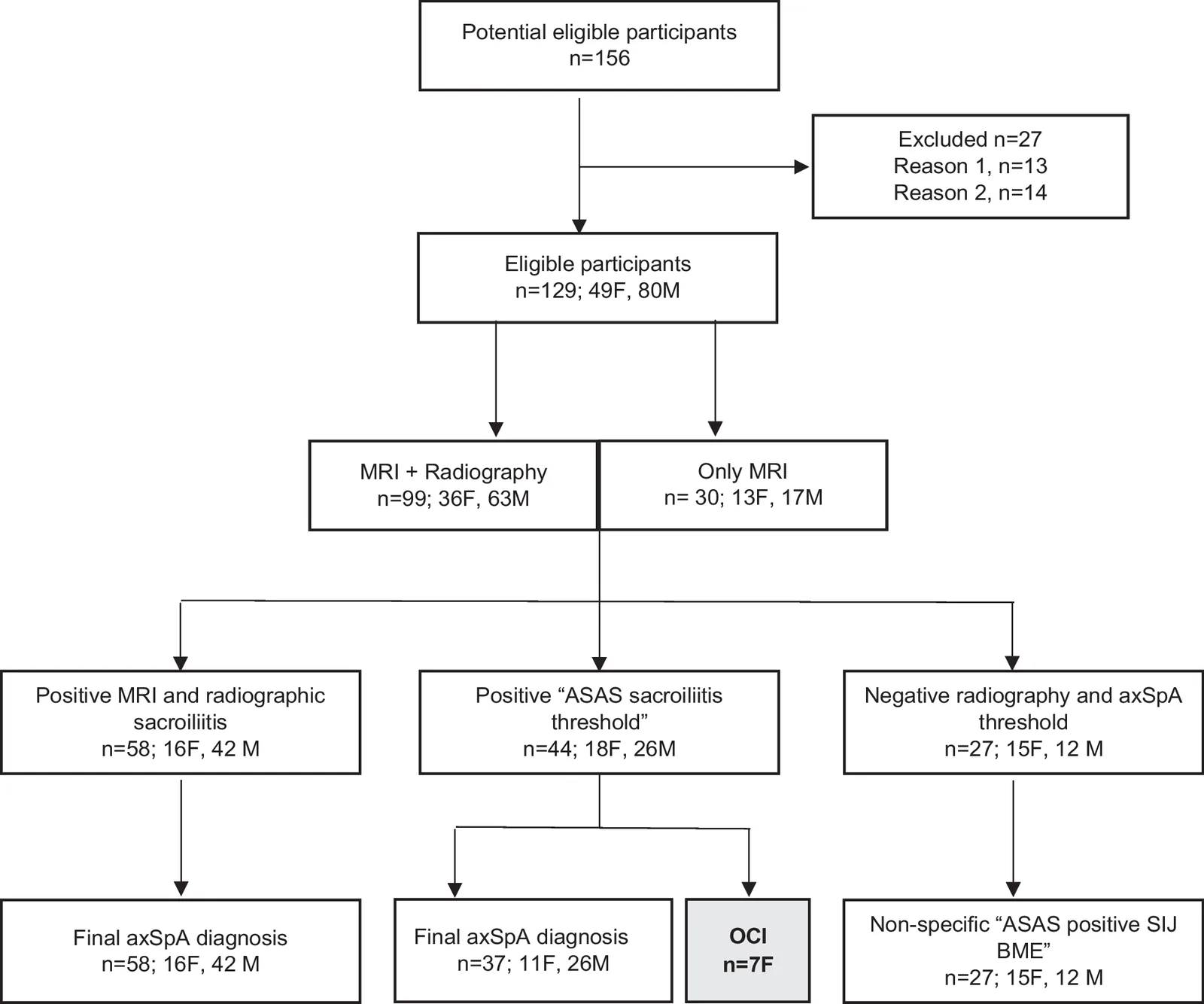
STARD flow diagram for inclusion and exclusion of study participants and imaging findings in the whole cohort. Positive “ASAS sacroiliitis threshold” = Data-driven MRI lesion threshold with positive predictive values of at least 95% for a clinical diagnosis of axSpA as proposed by the ASAS (Assessment of SpondyloArthritis International Society) MRI working group [19]; “ASAS positive SIJ BME” = BME fulfilling the ASAS classification criteria for active sacroiliitis [16, 20]; F women, M men

A consecutive cohort of patients aged ≥ 18 years diagnosed with axSpA at a secondary Department of Rheumatology, North Denmark Regional Hospital, Hjoerring, during the period 2012–2023 were retrospectively studied at follow-up. Participants were included in the study from the time of axSpA diagnosis (baseline) and followed until the 31th August 2023. Inclusion criteria were as follows: (1) Patients recorded with the international classification of diseases codes: M45. (r-axSpA, ankylosing spondylitis), M46.9 (inflammatory spondylopathy), M46.8 (inflammatory spondylopathy with extra-skeletal manifestations) and M46.1 (sacroiliitis, not elsewhere classified); (2) An active treatment course with either non-steroidal anti-inflammatory drugs or disease-modifying anti-rheumatic drugs at the Department of Rheumatology at commencement of this project August 2023; (3) Three or more appointments to evaluate the response to treatment; (4) Available Bath Ankylosing Spondylitis Disease Activity Index (BASDAI) score, at least at baseline; (5) MRI of the sacroiliac joint and entire spine at baseline, with concomitant radiography and/or computed tomography (CT) of the SIJ. Exclusion criteria were (1) less than three clinical evaluations and (2) lacking or insufficient MRI of the sacroiliac joint and/or the spine (Fig. 1).

### Study approval

The study was approved by the Danish National Committee on Health Research Ethics (number: 2309175) and was conducted in accordance with the Declaration of Helsinki II and subsequent revisions. Patient consent was waived due to the retrospective study design.

### Methodology and assessment of clinical data in the whole cohort

Initially, a list of potential study participants was obtained from the nationwide Danish registry of patients with rheumatic diseases, DANBIO, including information about CRP, BASDAI (0–100) and axSpA treatment(s). This was supplemented by a review of the electronic medical records with collection of the following data: sex, age at the initial diagnosis, age at onset of pain, body mass index (BMI), location of pain (lumbar spine and/or buttock, thoracic and cervical spine, and hips), information regarding physical demanding occupation, pregnancies/deliveries, familial disposition to axSpA, presence of HLA-B27 and associated peripheral arthritis, enthesitis, anterior uveitis, dactylitis, psoriasis and inflammatory bowel disease.

### Methodology and assessment of MRI, radiographs, and CT in the whole cohort

MRI of the SIJ was performed on 1.5 T MRI scanner and included semi-coronal T1-weighted and T1-weighted fat-suppressed (FS) sequences and a semi-axial short tau inversion recovery (STIR) sequence. MRI of the entire spine encompassed sagittal T1 and STIR or T2FS sequences.

All imaging material was analyzed by an experienced axSpA reader (AGJ) blinded to clinical data including a detailed as well as a global assessment of changes. Examinations during the follow-up period were analyzed similarly, and a final imaging diagnosis was established based on all findings.

The SIJ MRIs were assessed regarding subchondral BME and structural lesions (erosions, subchondral sclerosis and fat deposition, backfill and ankylosis) according to the SPARCC (Spondyloarthritis Research Consortium of Canada) MRI SIJ Structural Score using internationally accepted definitions [16–18]. The SIJ MRI changes were classified in accordance with data-driven MRI lesion thresholds with positive predictive values of at least 95% for a clinical diagnosis of axSpA as proposed by the ASAS (Assessment of SpondyloArthritis International Society) MRI working group in 2021, consisting either of BME highly suggestive of axSpA in at least 3 consecutive SIJ slices; erosions occurring in at least 3 SIJ scoring areas (with the joint divided into quadrants) or erosion at the same location in 2 consecutive slices; alternatively 5 areas with fat lesions, fat lesions at the same location in 3 consecutive slices or presence of a deep fat lesion (depth > 1 cm) [19]. Assessment of the fulfilment of ASAS classification criteria for active sacroiliitis consisting of at least two BME lesions on one MRI slice or BME on two consecutive slices was also recorded [16, 20] as the criteria sometimes were used clinically despite being classification criteria.

The spinal MRI assessment included signs of axSpA activity and structural changes in accordance with ASAS definitions [21] as well as vertebral endplate edema and fat deposition consistent with Modic changes [22], Pfirrmann grading of disc degeneration in the lumbar and cervical region [23], and disk protrusion according to Fardon [24].

SIJ radiographs and/or CT including the SIJ was obtained at baseline in 106 participants (42 women, 64 men). SIJ radiographs (available in 36 women, 63 men) were obtained as supine pelvic radiographs or angulated special view of the SIJs (Ferguson view). They were assessed in accordance with the Modified New York (mNY) criteria for ankylosing spondylitis [25]. Available CT of the SIJs was reconstructed in a straight coronal orientation, corresponding to a “pelvic radiograph”, and in a semi-coronal and semi-axial orientation corresponding to the slice orientations obtained by MRI, and was used to support structural MRI changes, especially when radiography was missing. The CTs were assessed for erosion, subchondral sclerosis, osteophytes, and joint space alteration.

The global assessment to determine whether the SIJ and spinal MRI findings and radiography/CT of the sacroiliac joint were indicative of axSpA (yes/no) was based on the presence and pattern of active inflammatory and structural changes with the reader indicating the confidence level on a scale from −5 (fully confident that it was not axSpA) to +5 (fully confident that it was axSpA).

Common SIJ differential diagnoses were systematically recorded by the primary investigator (AGJ), primarily to detect OCI changes defined as manifest well-demarcated subchondral iliac sclerosis with a depth ≥ 5 mm and absence of erosions, but non-specific BME located to SIJ load zones without well-demarcated subchondral iliac sclerosis, atypical anatomical morphology and lumbosacral transitional vertebra were also recorded [1].

The SIJ MRIs and radiographs/CTs of patients deemed to have OCI by the primary reader were sent digitally to a second experienced radiologist (AZ), who often performed secondary opinion evaluations. Blinded to clinical information, she was asked to determine whether MRIs and radiographs/CTs were indicative of axSpA, including a confidence level from −5 to + 5.

Finally, in consensus meetings between rheumatologist and radiologists, clinical, biochemical, and imaging findings were compared to confirm the presence of OCI.

The reference standard regarding presence or absence of axSpA in the whole cohort was likewise based on a combination of clinical, biochemical and imaging findings at the end of the study period in accordance with Standards for Reporting of Diagnostic Accuracy Studies (STARD) 2015 [26] (Fig. 1).

### Methodology and assessment of SIJ changes in patients with OCI

A detailed analysis of SIJ imaging features in patients with OCI was performed independently by two investigators (AGJ/RMK), including the following radiographic and CT changes: location of sclerosis and its maximal depth and the presence of erosions, osteophytes and joint space alteration. The MRIs were assessed for the same structural parameters in addition to the presence of subchondral BME and fat deposition, BME and/or fat deposition adjacent to the periphery of sclerotic lesions, BME adjacent to the periphery of subchondral fat deposition and fat deposition within sclerotic iliac areas. The location of sclerosis and fat deposition was assessed using a 2-plane MRI method with simultaneous assessment of semi-coronal and semi-axial images [27]. Based on the semi-axial images, the cartilaginous joint compartment was cranio-caudally divided into three joint regions (upper, middle and lower) including any slice, where at least one of the cartilaginous joint compartments was clearly visible for ≥ 1 cm in extent on ≥ 1 side. The number of semi-axial slices with sclerosis or fat deposition in the respective regions was counted and to adjust for differences in joint size, a relative sum score was calculated by dividing the absolute score by the number of semi-axial slices in the joint region and, for convenience, multiplied by 10. Anterior and/or posterior location of sclerosis was assessed based on the semi-axial slices.

The depth of sclerosis by MRI, radiography and CT, and the depth of subchondral sacral fat deposition by MRI were reported as mean values of the two assessors, whereas the location of sclerosis and presence of subchondral BME and fat deposition, BME and fat lesion adjacent to the periphery of sclerotic lesions, BME adjacent to the periphery of subchondral sacral fat deposition, and fat deposition within sclerotic areas were reported based on consensus between the two assessors.

### Data management and statistical analyses

Data of the whole cohort were entered directly into a customized REDCap (Research Electronic Data Capture) database [28]. Continuous variables were presented as medians with interquartile ranges (IQR, 25th–75th percentile) or means with standard deviations (SD) and ranges as appropriate whereas categorical variables were presented as proportions. Comparisons between groups were performed using Mann-Whitney *U* tests and Fisher’s exact tests. In patients with OCI inter-reader concordance for continuous MRI variables (depth of sclerosis and sacral fat deposition and their scores) was determined using intraclass correlation coefficient (ICC) values with 95% confidence intervals (95% CI) based on a 2-way, mixed-effects, single-measure model with individual absolute agreement using Stata version 15.1 [29].

## Results

A total of 129 patients were included in the main retrospective follow-up study, 49 women and 80 men, with a median age of 41 and 35 years and median symptom duration of 3.4 and 5.2 years at the baseline, respectively. Median BASDAI was 48 for both genders. The median follow-up period was 3.3 and 3.1 years, respectively. Demographic, clinical, and biochemical findings of the whole cohort are presented in Table 1.

**Table 1:** Demographic, clinical, and biochemical findings in the patients with OCI (cases) and the whole cohort

	The whole cohort Median values (IQR)/proportions		Cases						
	Male	Female	1	2	3	4	5	6	7
	*N* = 80	*N* = 49							
AxSpA diagnosis, year	2016 (2012–2019)	2018 (2013–2020)	2012	2016	2018	2020	2021	2022	2022
Clinical findings at baseline									
Age, years	35 (28–44)	41 (32–49)	31	32	35	36	33	47	41
Age at onset of pain	25 (21–34)	31 (24–44)	29	29	30	11	32	35	19
Symptom duration at baseline, years,	5.2 (1–10.7)	3.4 (2.0–9.4)	2	3	5	25	2	12	22
Pain location	N/A	N/A	l/b, t	l/b, h	l/b, c	l/b, t	l/b	l/b, t	l/b, c
BASDAI (0–100)	48 (26–63)	48 (32–63)	32	29	58	36	54	74	64
Elevated CRP due to axSpA	47.9%	26.7%	n	n	n	y	n	n	n
HLA-B27 positive	82.5%	55.1%	y	y	n	n	n	n	n
IBD	5.1%	20.4%	n	n	y	n	y	n	y
Peripheral arthritis	27.5%	26.5%	n	n	y	n	n	n	n
Enthesitis	27.1%	20.9%	n	n	?	y	y	y	y
Anterior uveitis	21.5%	8.3%	n	n	n	?	n	n	n
Dactylitis	7.6%	6.1%	n	n	y	n	n	n	n
Psoriasis	10.1%	14.3%	n	n	n	n	n	n	n
Familial disp. axSpA	16.2%	28.9%	n	n	?	?	n	n	n
BMI (kg/m^2^)	28 (25–31)	25 (23–28)	27	23	17	37	24	29	23
Preceding childbirths*, no	N/A	2 (1–2)	1	1	3	1	2	0	2
Time since childbirth(s), years	N/A	8.5 (5–12)	1.5	3	6, 8.5, 11	14	3, 4	n	9, 15
Physical demanding occupation	32.5%	8.2%	n	?	n	n	n	n	n
Follow-up period, years	3.1 (2.1–6.4)	3.3 (1.4–5.8)	11	7	5	3	2	1	1

*N*, number; *N/A*, non-applicable; *IQR*, interquartile range 25–75; *l/b*, lumbar spine and/or buttock; *h*, hip; *c*, cervical spine; *t*, thoracic spine; *BASDAI*, Bath Ankylosing Spondylitis Disease Activity Index; *BMI*, Body Mass Index; *CRP*, C-reactive protein; *disp.*, disposition; *IBD*, inflammatory bowel disease; *proportions*, proportion of patients; *n*, no; *y*, yes,? = unknown

*After the 20^th^ pregnancy week.

SIJ changes exceeding MRI thresholds for axSpA occurred in 102 participants (34 women, 68 men); 58 of them had radiographic changes fulfilling the mNY criteria and confirmed axSpA by global imaging assessment supported by their clinical features. MRI thresholds were exceeded in 44 participants (18 women, 26 men) with clinical findings consistent with axSpA, but the presence of axSpA could not be confirmed by global imaging assessment in 7 women who had changes consistent with OCI (Fig. 1). The remaining 27 participants (15 women, 12 men) all had low back pain and SIJ BME fulfilling the ASAS classification criteria [16, 20], but were by the global imaging assessment deemed not to have axSpA (confidence levels <−1). Their SIJ BME was related to load or degenerative changes in 7 women/5 men, atypical anatomical morphology in the form of accessory joints or dysmorphic joint in 6 women/6 men and lumbosacral transitional vertebra in 2 women/1 man (Fig. 1). The sensitivity and specificity of the combined clinical and MRI findings in relation to axSpA were 100% and 79%, respectively.

**Fig. 2:**
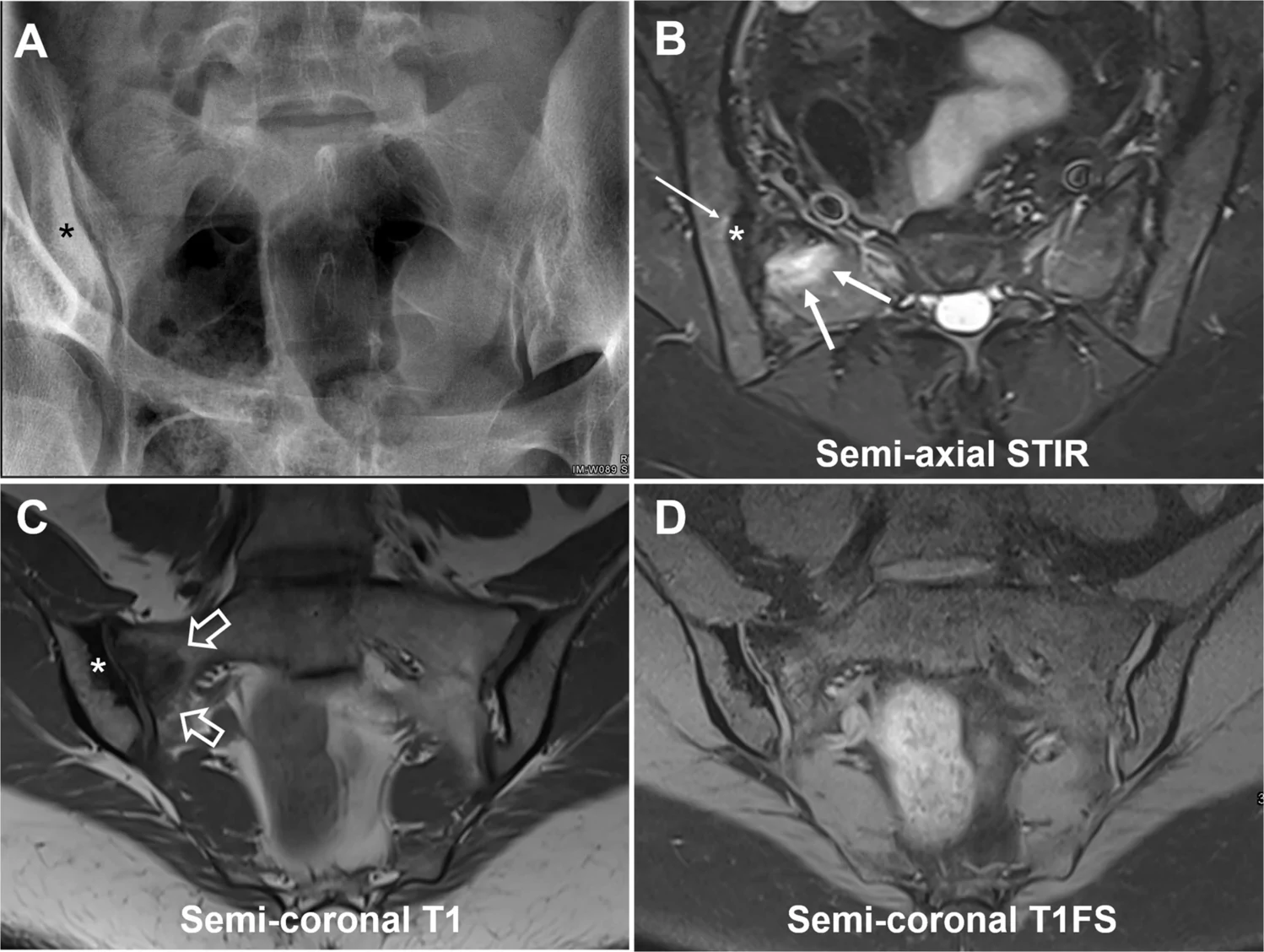
33-year-old woman with a clinical diagnosis of axSpA, Case 5, initial examinations, **A** anterior-posterior angulated radiograph of the sacroiliac joints (SIJs) and **B**–**D** MRI, semi-axial STIR image **B**, and semi-coronal T1-weighted image **C** and T1-weighted with fat saturation (FS) **D**. Radiographically, there is right-sided iliac sclerosis mainly located at the upper and middle part of the joint, also visible by MRI (asterisks). MRI in addition shows bone marrow edema (BME) at the right SIJ predominantly located anteriorly in the sacrum (arrows) with a slim brim of surrounding fat deposition (open arrows) extending to the subchondral area anteriorly (not shown). There is also a brim of BME peripherally to the iliac sclerosis (slim arrow)

In the seven patients deemed to have OCI, the primary reader indicated a confidence level of −5 to −1 (median −4) whereas the confidence levels of the second imaging investigator were −5 to 0 (median −5) (Table 2). The largest discrepancy of −4 occurred in a woman with pronounced sacral BME and only moderate iliac sclerosis (Fig. 2). Both investigators had a low confidence level (−1 and 0, respectively) in a 47-year-old woman with slight right-sided OCI changes. However, at the clinical and imaging consensus meetings, it turned out that she had been overweighted for many years with a BMI around 30 kg/m^2^, had characteristic radiographic right-sided OCI changes 12 years previously, and did not develop erosions consistent with an OCI diagnosis.

**Table 2:**
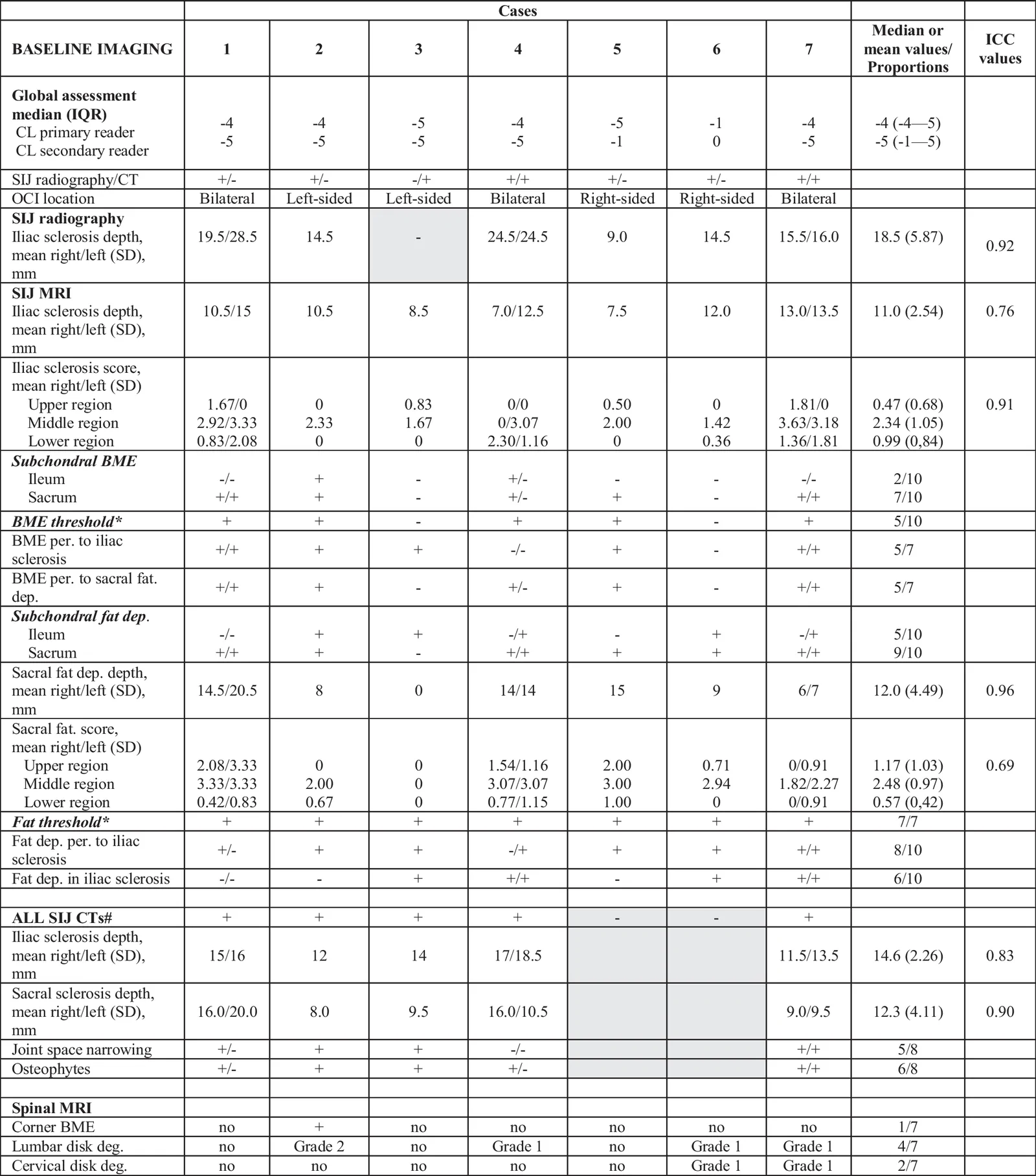
Imaging findings in the patients with OCI

+  = present; - = absent; *CL*, confident level; *IQR*, interquartile range 25-75; *SD,* standard deviation; *SIJ,* sacroiliac joint; *dep.,* deposition; *per.,* peripheral;* Disk deg.*, disk degeneration graded according to Pfirrmann 2006 [23]; *proportions*, proportion of joints/patients; *ICC, *intraclass correlation coefficient. *Data-driven MRI lesion threshold with positive predictive values of at least 95% for a clinical diagnosis of axSpA [19]

#Represent CT data at baseline and during follow-up

The main demographic, clinical, and biochemical findings in the 7 women with an OCI consensus diagnosis appear in Table 1*.* Their age at baseline varied between 31 and 47 years (median 35 years) and the duration of symptoms between 2 and 25 years (median 5 years). The median BASDAI was 54, but periods with inflammatory pain characteristics (pain at night, morning stiffness, improvement with exercise, but not with rest) occurred only in two patients (cases 4 and 7). BMI ranged between 17 and 37 kg/m^2^ (median 24), two women were HLA-B27 positive, and six had preceding deliveries 1.5–15 years previously (median 7). The follow-up period was 1–11 years (median 3 years). The time since last childbirth was significantly shorter in the OCI women compared with the remaining women, median values 53.3 (IQR 36.6–71.4) and 118.4 (IQR 82,4–161.6) months, respectively, *p* = 0.02. No other statistical differences were detected by comparing the OCI women with the remaining women or all remaining patients in the whole cohort.

### Imaging findings in patients with OCI

**Fig. 3:**
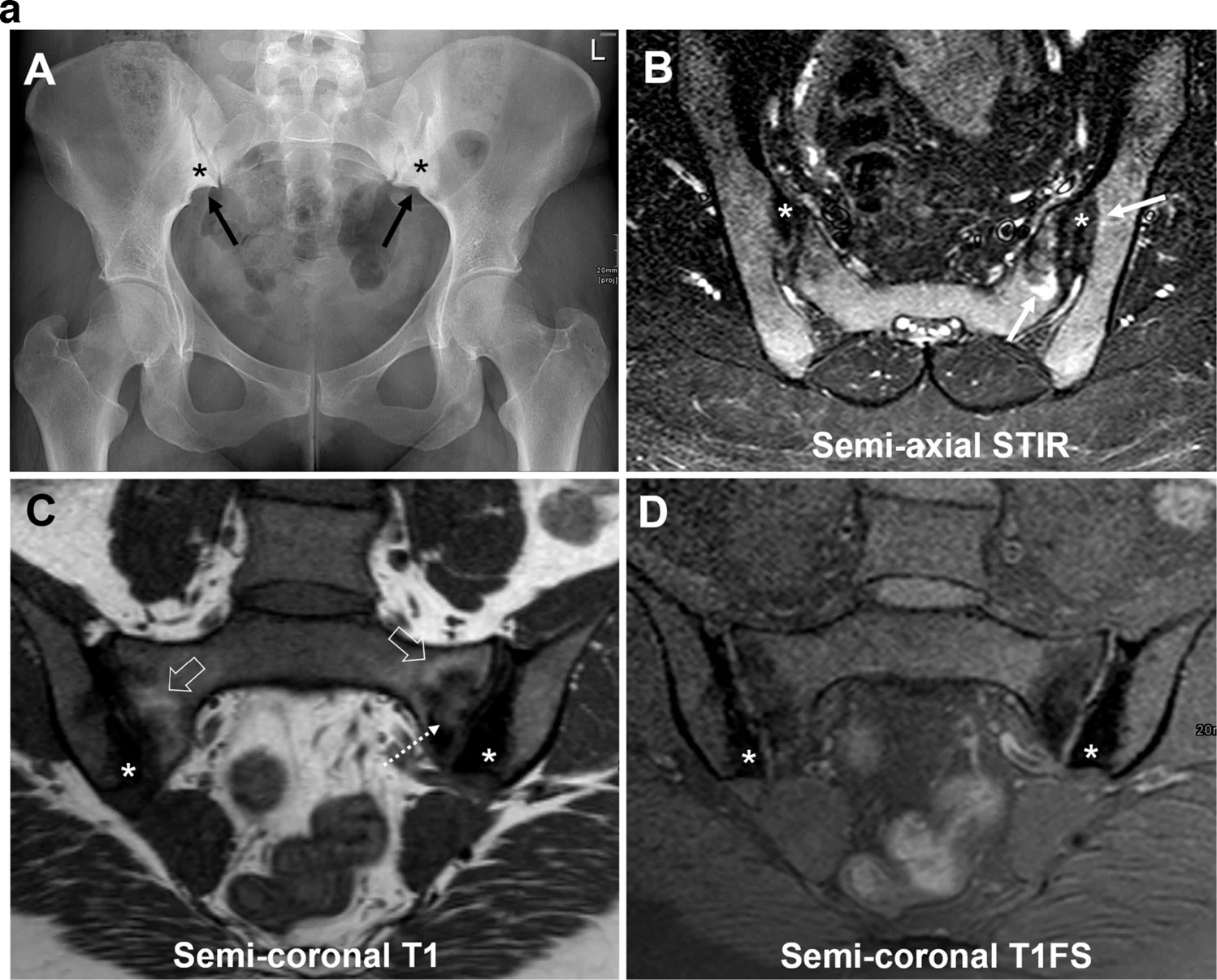

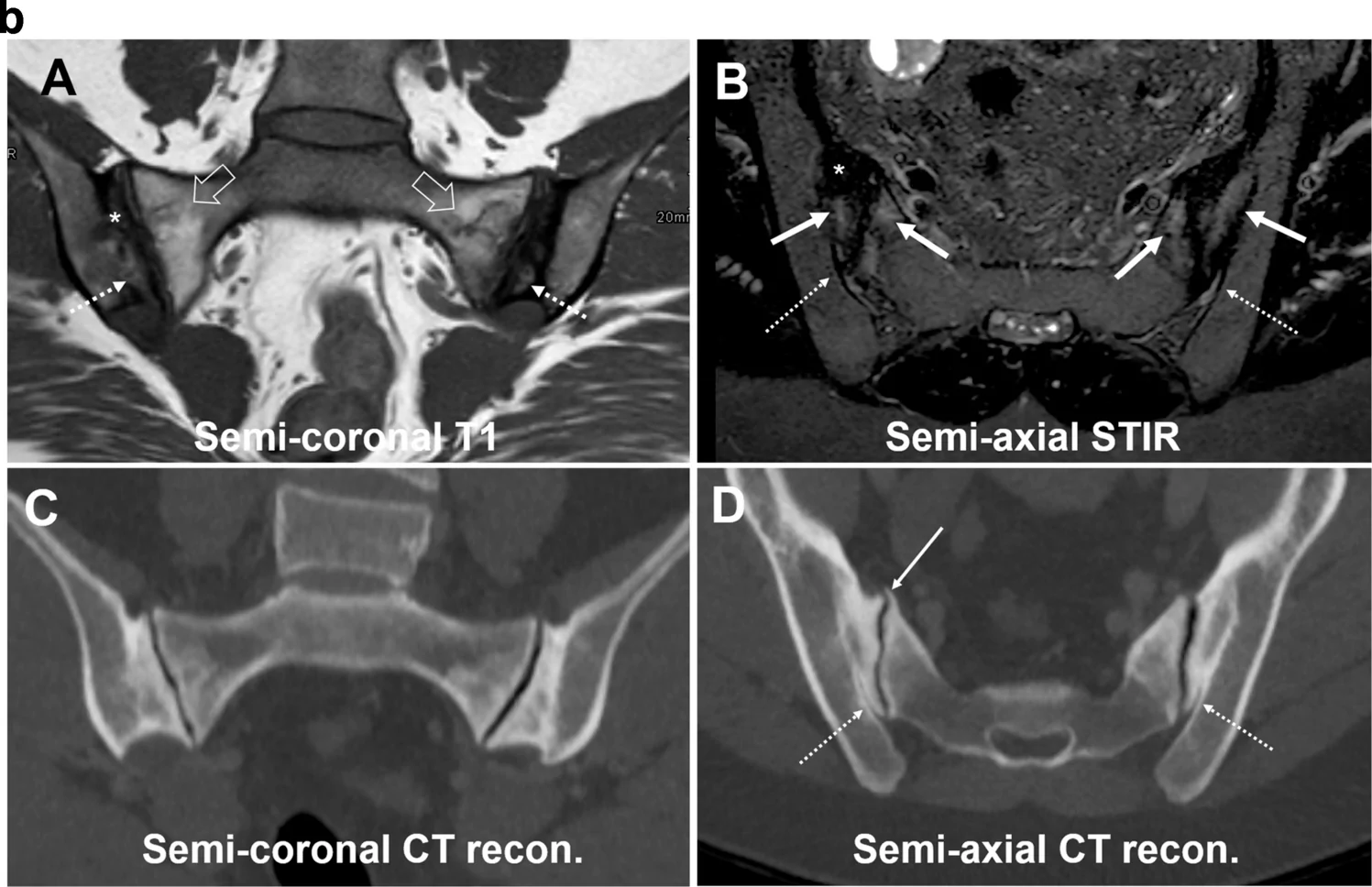
31-year-old woman with a clinical diagnosis of axSpA, Case 1. **a** Initial examinations, **A** anterior-posterior pelvic radiograph and (B–D) MRI, semi-axial STIR image **B**, and semi-coronal T1-weighted image **C** and T1-weighted with fat saturation (FS) **D**. Radiographically, there is bilateral triangular iliac sclerosis at the lower part of the sacroiliac joints (SIJs) and paraglenoid sulcus formation (black arrows). On MRI, there is bilateral homogenous iliac sclerosis (asterisks) with a peripheral brim of bone marrow edema (BME) at the left SIJ. More pronounced BME is seen in the sacrum peripheral to a low signal intensity area consistent with a mixture of sclerosis and fat deposition, but also extending to the subchondral area (arrows). There is bilateral fat deposition in the sacrum (open arrows) surrounding subchondral areas with a mixture of sclerosis and fat deposition (dashed arrow). **b **MRI 10 years later when the clinical axSpA diagnosis was dubious, **A** semi-coronal T1-weighted and **B** semi-axial STIR images show persistence of iliac sclerosis (asterisks), but with dispersed areas of fat deposition (dashed arrows). There is now scattered BME at both SIJs (arrows), and the fat deposition in the sacrum has markedly increased on both sides (open arrows) replacing most of the previously sclerotic areas. CT further 1 year later, **C** semi-coronal and **D** semi-axial reconstructions, shows no sign of erosions, but bilateral vacuum phenomenon and right-sided joint space narrowing with concomitant osteophyte formation, especially anteriorly (slim arrow). There is bilateral increased density in the subchondral bone consistent with sclerosis, but not homogeneous dense consistent with areas of both BME and fat within the sclerosis on MRI. There is bipartite iliac bone plate (slim dashed arrows) best visualized by CT

**Fig. 4:**
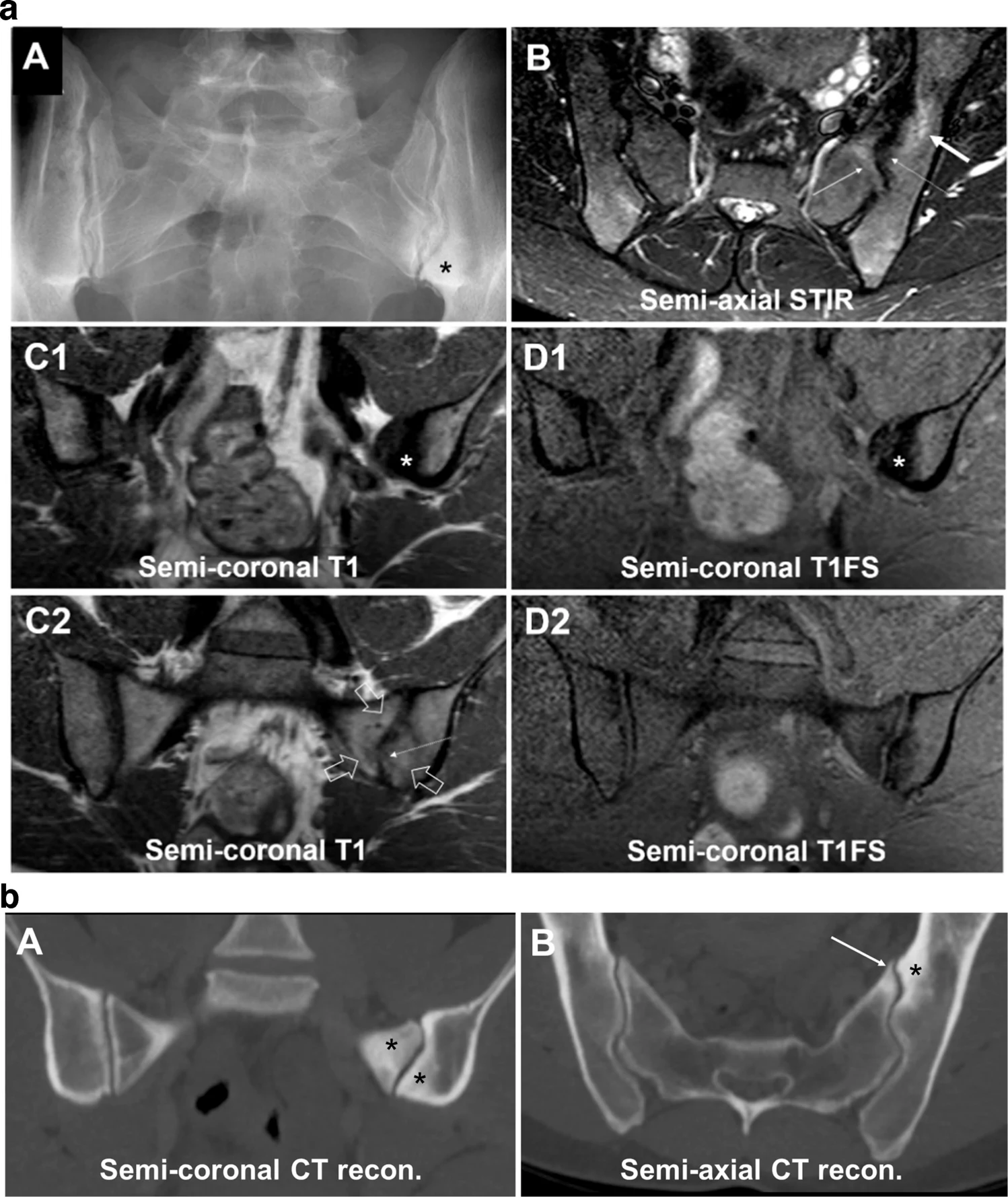
32-year-old woman with a clinical diagnosis of axSpA, Case 2.** a** Initial examinations, **A** angulated anterior-posterior view of the sacroiliac joints (SIJs) and **B**–**D** MRI, semi-axial STIR image **B**, and semi-coronal T1-weighted images (C1,2) and T1-weighted with fat saturation (FS) (D1,2), the two most anterior slices. Radiographically, there is triangular iliac sclerosis at the lower part of the left SIJ (asterisk), also visible on the most anterior MR slices (C1 and D1) (asterisks). MRI in addition shows bone marrow edema (BME) at the left SIJ located peripheral to the iliac sclerosis (arrow) and also subchondral in the sacrum (slim arrow). There is fat deposition in the sacrum and also in the ilium posteriorly to the sclerotic area (open arrows). The joint configuration is dysmorphic with an iliac prominence into the sacral joint facet (slim dashed arrows). **b **CT 1 year later, still with a clinical diagnosis of axSpA, **A** semi-coronal and **B** semi-axial reconstructions show no sign of erosions, but left-sided joint space narrowing with concomitant minor osteophyte formation (slim arrow) and subchondral sclerosis on both side of the joint (asterisks).

Baseline examinations included SIJ radiography in 6 women and CT in one woman in addition to the MRI. Two further women had an available CT including the SIJ at baseline performed due to other indications. OCI changes occurred in 10 SIJs by MRI and radiography/CT. Three women had bilateral OCI changes (Fig. 3), two right-sided and two left-sided changes (Table 2, Figs. 2 and 4). Follow-up imaging was performed in three women after 1–11 years. One woman obtained three SIJ MRIs and one CT examination (Case 1, Fig. 3), and two a CT examination (Cases 2 and 7).

**Fig. 5:**
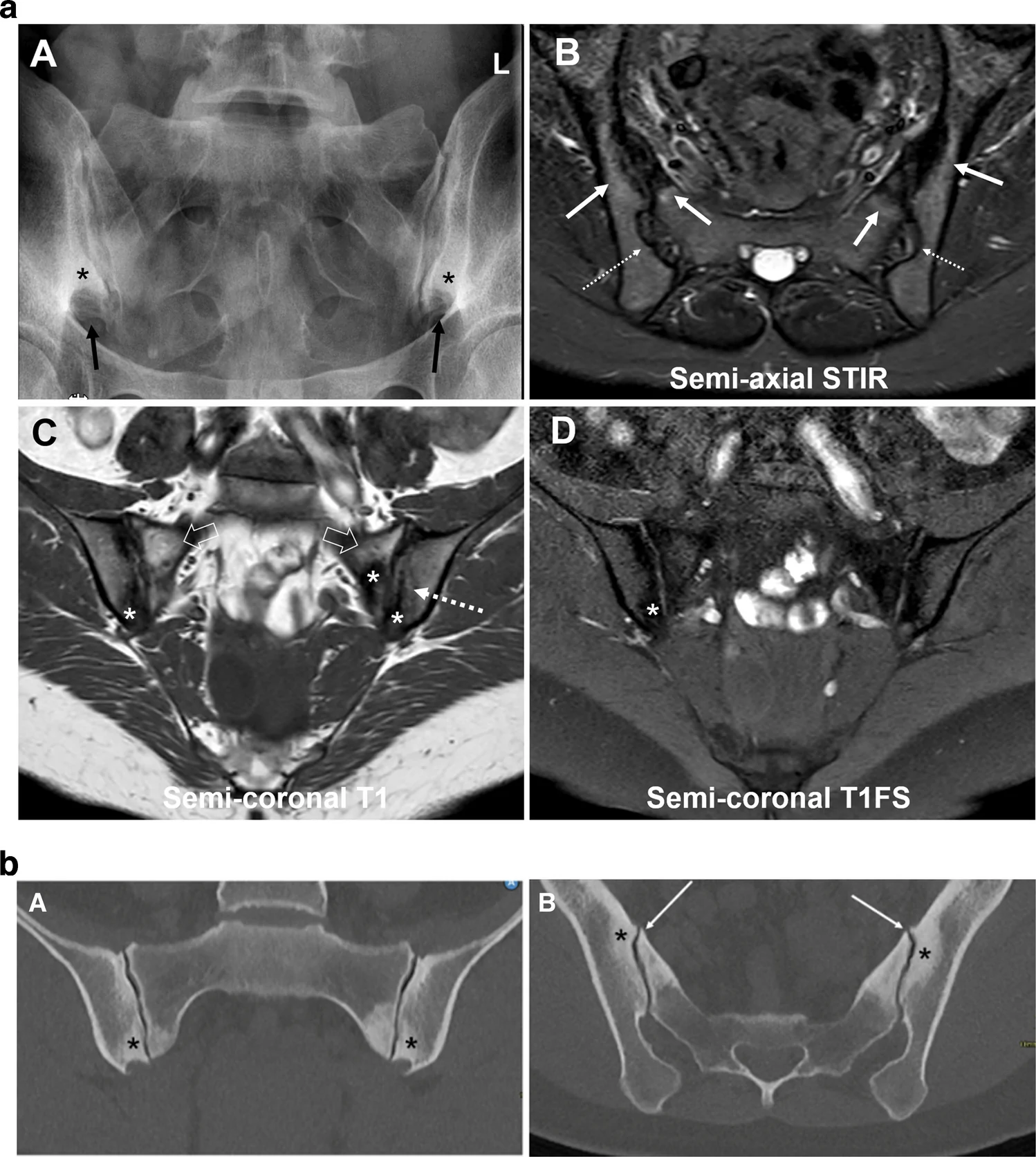
41-year-old woman with a clinical diagnosis of axSpA, Case 7. **a** Initial examination, **A** anterior-posterior angulated radiograph of the sacroiliac joints (SIJs) and **B**–**D**) MRI, semi-axial STIR image **B**, and semi-coronal T1-weighted image **C** and T1-weighted with fat saturation (FS) **D**. Radiographically, there is bilateral triangular iliac sclerosis at the lower part of the SIJ (asterisks) with concomitant paraglenoid sulcus formation (black arrows). On MRI there is manifest bilateral iliac sclerosis and also a sclerotic area in the sacrum at the left SIJ (asterisks) with surrounding fat deposition (open arrow). There is concomitant bone marrow edema peripheral to the iliac sclerosis as well as peripheral to an anterior sacral area with low signal intensity on the STIR image consistent with fat deposition intermingled with sclerosis (arrows). A small area with fat deposition is seen within the left-sided iliac sclerosis (dashed arrow). Anatomical variation with crescent-like iliac bone plate at the right SIJ (long slim dashed arrow) and semicircular defect at the left SIJ (short slim dashed arrow) is seen on the axial slice. **b** CT 1 year previously more clearly demonstrated the iliac sclerosis (asterisks) with concomitant sacral sclerosis in addition to relative narrow joint spaces with small osteophytes anteriorly (slim arrows).

None of the 7 women fulfilled the mNY criteria or had CT detectable erosions. The detailed analysis of radiographs of 9 OCI joints revealed subchondral iliac sclerosis with a mean depth of 18.5 mm (Table 2). The sclerosis was in five women located to the lower part of the joint with a triangular appearance (Figs. 3, 4, and 5), but it appeared located to the middle joint portion in two women obtaining angulated SIJ radiographs, which influenced the location of sclerosis (Fig. 2). Sacral sclerosis and osteophytes or certain joint space narrowing were not detected, but paraglenoid sulcus formation occurred in two women [30] (Figs. 3 and 5).

The mean depth of iliac sclerosis by MRI was 11.0 mm at baseline. The sclerosis was most frequent at the middle joint region where the mean sclerosis sum score was 2.34 compared with mean scores of 0.47 and 0.99 in the upper and lower joint regions, respectively (Table 2). The sclerosis was in all OCI joints located anteriorly but extended to posterior joint areas in five joints. It exhibited well-demarcated border laterally and had homogenous low signal intensity on all sequences except the occurrence of small fat deposition within the sclerosis, observed in six OCI joints (Table 2, Fig. 5). Concomitant sacral sclerosis located to the middle joint region was detected in six OCI joints with a mean depth of 7.0 mm (Figs. 3 and 5).

All 7 women had BME at the SIJs, but only five fulfilled the data-driven threshold for subchondral BME consistent with axSpA [19] (Table 2). Their subchondral BME was primarily located in the sacrum (Figs. 2, 3, and 4) but was in two women accompanied by small areas of subchondral BME in the ilium (Table 2). However, the data-driven fat threshold was fulfilled in all 7 women. The subchondral fat deposition predominated in the sacrum where it had a mean depth of 12.0 mm and was most extensive at the middle and upper joint region with mean scores of 2.48 and 1.17, respectively, compared with 0.57 for the lower joint region (Table 2, Figs. 3 and 5).

BME with a continuous appearance located peripheral to the iliac sclerosis and peripheral to sacral fat deposition each occurred in 7 OCI joints (five women) and fat deposition peripheral to the iliac sclerosis occurred in 8 OCI joints (all 7 women) (Table 2, Figs. 2, 3, 4, and 5). Erosions, osteophytes and joint space narrowing were not detected by MRI.

The mean depth of iliac sclerosis on all available CTs of 8 OCI joints (5 women) was 14.6 mm with no depth difference at baseline and during follow-up, mean values 14.9 mm and 14.3 mm, respectively. Concomitant sacral sclerosis occurred in all 8 OCI joints with a mean depth of 12.3 mm. Osteophytes were present in five OCI joints, most pronounced anteriorly (Figs. 3, 4, and 5), and joint space narrowing was seen in five OCI joints (Figs. 3, 4, and 5), but erosions and ankylosis did not occur.

Atypical anatomical SIJ morphologies were present in all women. Bipartite iliac bone plate was seen in five women (cases 1,3,4,5 and 6, Fig. 3), dysmorphic joint configuration in two (cases 2 and 7, Fig. 4), crescent-like iliac bone plate in two (cases 5 and 7, Fig. 5), semicircular defect in two (cases 4 and 7, Fig. 5) and iliosacral complex in one woman (case 2), but accessory sacroiliac joints were not detected.

None of the patients had active inflammatory or structural spinal MRI changes consistent with axSpA, but one patient had slight BME at vertebral corners with an appearance consistent with degenerative changes. Slight lumbar disk degeneration (grades 1–2) occurred in four women, in two accompanied by slight disk degeneration in the cervical spine (Table 2), but endplate edema or fat deposition and disk protrusions did not occur.

### Reliability of detected MRI lesions in patients with OCI

Interreader agreement between the two readers for the detected MRI lesions in patients with OCI, depth of iliac sclerosis by MRI, radiography and CT and sacral fat deposition and their MRI scores was moderate for sacral fat score, but good or excellent for the other parameters (Table 2).

## Discussion

AxSpA was confirmed in 95 participants using our combination of clinical and MRI findings with supplementary global imaging assessment corresponding to a specificity of 79% which is in accordance with previous findings [31]. Seven women had MRI changes exceeding the threshold for axSpA but were by global assessment found to have OCI whereas 27 participants had low back pain and non-specific SIJ BME.

None of the seven women with OCI had radiographic structural changes consistent with r-axSpA according to the mNY criteria or erosions by MRI/CT. They all fulfilled data-driven MRI lesion thresholds with a positive predictive value (PPV) of at least 95% for a clinical diagnosis of axSpA, five based on both subchondral BME and fat deposition and two only based on fat deposition [19]. According to the ASAS definition, subchondral BME is required for classifying a patient as having active axSpA sacroiliitis [17]. BME as well as fat deposition peripheral to sclerosis were therefore not used as valid sacroiliitis features. There is not a corresponding 95% threshold for subchondral sclerosis which was reported to have a maximal PPV value of 91% [19].

Despite the present somewhat varying OCI MRI changes influenced by age, disease duration and BMI, the following observed characteristic features can facilitate the OCI diagnosis. The iliac sclerosis was predominantly located at the load-related anterior-middle joint areas. It had a relative sharp demarcation laterally and accompanying BME and fat deposition peripheral to the sclerosis were frequent. Manifest subchondral fat deposition in the sacrum was frequent, predominantly located to the middle joint area and occasionally with concomitant BME at the periphery, and subchondral BME predominated in the sacrum. Concomitant sacral sclerosis seems frequent, detected in six of the 10 OCI joints by MRI, but observed in all joints evaluated by CT. It is possible that the frequently occurring subchondral sacral fat deposition giving a high signal on T1-weighted images can obscure sacral sclerosis by MRI.

Some characteristic OCI MRI-features have previously been reported in a cross-sectional study and two case-control studies encompassing 27, 27, and 34 patients with OCI, respectively [7–9]. The most consistent finding was iliac sclerosis located anteriorly and frequently accompanied by BME, reported present in 48–93% of patients with OCI and usually located anteriorly. Our findings were in accordance with this. However, distinction between subchondral BME and BME peripheral to sclerotic areas has not previously been reported although BME located peripheral to sclerosis and displaying a continuous appearance, as seen in the current study, was observed in one study [7]. Fat deposition was only assessed in one of the previous studies where the prevalence and score of fat in the sacrum was higher than in the ileum, consistent with our findings [8].

The present mean sclerosis depth of 11 mm by MRI was slightly lower than the depth of 13 mm (range 7–22 mm) observed in a previous study [7], but our mean depth of 14.6 mm by CT was larger than the 11 mm (range 8–15 mm) observed in another group [9]. Different composition of study groups can probably explain these differences. The current sclerosis depths on radiographs and CT were larger than by MRI which probably is caused by the presence of fibrovascular material in the bone marrow causing increased X-ray attenuation by radiography/CT but giving rise to signals by MRI [32, 33].

The present detailed MRI findings in the OCI patients were not compared with axSpA changes, but a comparison was obtained in a previous case-control study encompassing 27 OCI (96.3% women) and 27 axSpA patients (33.3% women) matched 1:1 by back pain duration [8]. The patients with OCI were found to have higher prevalence and scores of sclerosis by MRI than axSpA patients whereas the prevalence and scores of BME were rather similar. Fat deposition was common both in OCI and in axSpA but with significantly lower scores in OCI than in axSpA, and erosions were infrequent in OCI. In nearly all OCI patients, the BME, sclerosis, and fat deposition were located to the anterior portion of the SIJ whereas the axSpA lesions were more dispersed [8]. In addition, the prevalence and scores of BME and fat deposition were in OCI higher in the sacrum than in the ileum whereas BME and fat deposition in axSpA were equally frequent in the ileum and sacrum. The current finding of subchondral BME and fat deposition predominantly in the sacrum is consistent with these findings and can be regarded a feature of OCI.

The CT changes in 34 OCI patients have been compared with an age- and sex-matched group of patients without SIJ disorders. The only significant differences observed were a higher frequency of osteophytes and geodes in the patients with OCI, but the sclerosis depths were generally higher in OCI than in the control patients, mean values 11 mm (8–15 mm) and 5 mm (4–6 mm), respectively, and sclerosis depths above 10 mm did not occur in the controls [9].

Except a shorter time since last childbirth, the clinical and biochemical findings of the current OCI women did not differ significantly from the remaining women or all other patients in the whole cohort. This may be due to the low number of OCI patients. In the previous case control study comparing OCI and axSpA patients matched 1:1 by back pain duration, OCI patients had a lower frequency of inflammatory back pain, HLA-B27 positivity (35.2% vs 80.0%), elevated CRP, peripheral arthritis, uveitis, dactylitis, heel enthesitis, and inflammatory bowel disease than axSpA patients [8].

Six of the present women had preceding pregnancy and childbirth, and the woman without preceding pregnancy was obese implying increased load to the SIJ. A frequent relation of OCI to pregnancy/delivery was also observed in previous studies [7–9] and supported by a frequent occurrence of subchondral sclerosis in postpartum women [6, 34–36], all indicating that hormonal changes can contribute to the development of OCI. However, the etiology and evolution of OCI is still speculative, especially as iliac sclerosis also can be seen due to mechanical load, e.g. related to scoliosis and hip dysplasia [10, 37], and load-related, mechanical as well as degenerative sclerotic changes are like OCI changes usually most pronounced in the anterior middle joint portion [27]. This indicates that load may play a part and the female predominance among individuals with OCI could partly be due to a different joint load in women compared to men caused by increased tilt of the pelvis. Also, the frequent occurrence of anatomical variations in females may influence the joint load [38]. Despite the presence of BME by MRI, inflammatory histopathologic changes have never been detected in OCI [32, 33]. It is possible that the BME in OCI is elicited by intraosseous strain and that OCI changes represent pronounced or atypical mechanical related SIJ pathologies, predominantly occurring in women but occasionally diagnosed in men and nulliparous women where it is often related to obesity or excessive load to the SIJ [7, 9, 10, 14]. The current and previously observed relative frequent occurrence of degenerative SIJ changes in OCI patients by CT in the form of osteophytes and joint space narrowing support that load is a pathogenetic factor [9]. MRI is not optimal for detecting load-related and/or degenerative SIJ changes. CT is in general superior to MRI and radiography for visualizing structural SIJ changes in the form of erosions, sclerosis and joint space alterations as well as osteophytes due to degenerative changes which are not always detectable by MRI and radiography [39–42]. The osteophytes detected by CT in 6 of 8 OCI joints in the current study were most pronounced anteriorly, an area only visualized on semi-axial STIR images which were not optimal for anatomical delineation and small osteophytes may vanish in the relative thick MR-slices compared to CT-slices [41]. By radiography, only osteophytes located to the inferior and superior joint margin can be detected but were not observed in the current patients. Also, radiographic joint space narrowing and sacral sclerosis were not observed. However, two women had paraglenoid sulcus formation, which seems related to mechanical load on the SIJ and previously observed together with OCI changes [30].

OCI MRI changes have, to our knowledge, only been evaluated longitudinally in one study where the sclerosis was seen to regress over time, whereas BME remained unchanged [9]. Regression of sclerosis was observed in one of the current women with available radiograph 12 years prior to inclusion but was not detected in the other patients. However, the occurrence of small fat deposition within sclerotic iliac areas indicates that the sclerosis gradually can resolve over time. Consistent with this, partly or complete resolution of radiographic sclerosis has been observed in 16 of 19 subjects followed for more than 8 years [13].

## Strengths and limitations

A strength of this study was uniform baseline MRIs and clinical follow-up of all participants as well as imaging assessment by a radiologist experienced in axSpA imaging. Nevertheless, only one radiologist assessed all examinations obtained in the main cohort, introducing potential single-observer bias although all patients with OCI were evaluated in detail by two experienced radiologists blinded to clinical information. A similar detailed analysis of axSpA patients was not performed for comparison. The OCI material was limited, but gives insight into the MRI, radiographic, and CT appearances of OCI changes. The main cohort was also limited, consisting of 129 patients treated at a single center. However, each patient was followed and had at least three appointments.

In conclusion, MRI OCI changes can vary somewhat being influenced by age, disease duration, pregnancies/deliveries and BMI, but the following characteristic features were detected: the iliac sclerosis in OCI was predominantly located at the load-related anterior-middle area of the SIJ and accompanying BME and/or fat deposition adjacent to the periphery of iliac sclerosis was frequent. Subchondral BME and fat deposition predominated in the sacrum and erosions were absent.

## Data Availability

Data are available from the first and last author (Dr. Anne Grethe Jurik and Dr. Asta Linauskas upon request.

## Abbreviations

ASAS: Assessment of SpondyloArthritis International Society
axSpA: Axial spondyloarthritis
BASDAI: Bath Ankylosing Spondylitis Disease Activity Index
BME: Bone marrow edema
BMI: Body mass index
CT: Computed tomography
CRP: C-reactive protein
FS: Fat-suppressed
HLA-B27: Human leukocyte antigen subtype B27
OCI: Osteitis condensans ilii
STIR: Short tau inversion recovery
SIJ: Sacroiliac joint
mNY criteria: Modified New York criteria
